# Association between serum sphingolipids and eudaimonic well-being in white U.S. adults

**DOI:** 10.1038/s41598-021-92576-3

**Published:** 2021-06-23

**Authors:** Loni Berkowitz, Marcela P. Henríquez, Cristian Salazar, Eric Rojas, Guadalupe Echeverría, Gayle D. Love, Attilio Rigotti, Christopher L. Coe, Carol D. Ryff

**Affiliations:** 1 Department of Nutrition, Diabetes and Metabolism, Center of Molecular Nutrition and Chronic Diseases, School of Medicine, Pontificia Universidad Católica de Chile, Marcoleta 328, Santiago, Chile; 2 ELSA Clinical Laboratory, IntegraMedica, Bupa, Santiago, Chile; 3 Department of Clinical Laboratory, School of Medicine, Pontificia Universidad Católica de Chile, Santiago, Chile; 4 Institute on Aging, University of Wisconsin-Madison, Madison, WI USA

**Keywords:** Quality of life, Lipidomics, Psychology

## Abstract

Emerging research has linked psychological well-being with many physiological markers as well as morbidity and mortality. In this analysis, the relationship between components of eudaimonic well-being and serum sphingolipids levels was investigated using data from a large national survey of middle-aged American adults (Midlife in the United States). Health behaviors (i.e., diet, exercise, and sleep) were also examined as potential mediators of these relationships. Serum levels of total ceramides—the main molecular class of sphingolipids previously associated with several disease conditions—were inversely linked with environmental mastery. In addition, significant correlations were found between specific ceramide, dihydroceramide, and hexosylceramides species with environmental mastery, purpose in life, and self-acceptance. Using hierarchical regression and mediation analyses, health behaviors appeared to mediate these associations. However, the link between ceramides and environmental mastery was partially independent of health behaviors, suggesting the role of additional mediating factors. These findings point to sphingolipid metabolism as a novel pathway of health benefits associated with psychological well-being. In particular, having a sense of environmental mastery may promote restorative behaviors and benefit health via improved blood sphingolipid profiles.

## Introduction

The World Health Organization defines health as a state of full mental, physical and social well-being, rather than just the absence of disease. Whereas poor psychological functioning (e.g., anxiety, depression, anger) can increase the risk for physical diseases^1^, emerging evidence has consistently identified a protective relationship between psychological well-being (PWB) and physical health^2,3^.

PWB is considered a multidimensional construct with distinct components. Hedonic well-being typically refers to the pursuit of pleasure, life satisfaction, and happiness. Eudaimonic well-being, in contrast, addresses fulfilling one’s potential and identifying meaningful life pursuits^4,5^. The theory-based formulation of eudaimonic well-being includes six domains: autonomy, environmental mastery, personal growth, positive relations with others, purpose in life, and self-acceptance^4^.

Growing evidence supports the positive benefits of both types of PWB on physical health outcomes^2,6^. They have been associated with restorative health behaviors and improved physiological functioning^7^. Specifically, eudaimonic well-being has been inversely associated with unhealthy behaviors, such as excessive alcohol consumption^8^, physical inactivity^9^, poor sleep quality^10^, and unhealthy diet^11^ as well as risk for cardiometabolic disorders^2,7,12^. In this context, PWB has been linked cross-sectionally and prospectively with favorable traditional blood lipid indices. For instance, persistently high levels of PWB predicted higher HDL cholesterol and lower triglyceride levels during a 9–10 year follow-up^13^. Although this lipid pattern appears to protect against atherosclerotic cardiovascular disease (ASCVD), there is a need to consider additional lipid measures as predictors of low versus high atherogenic propensity and risk for other chronic conditions.

**Figure 1:**
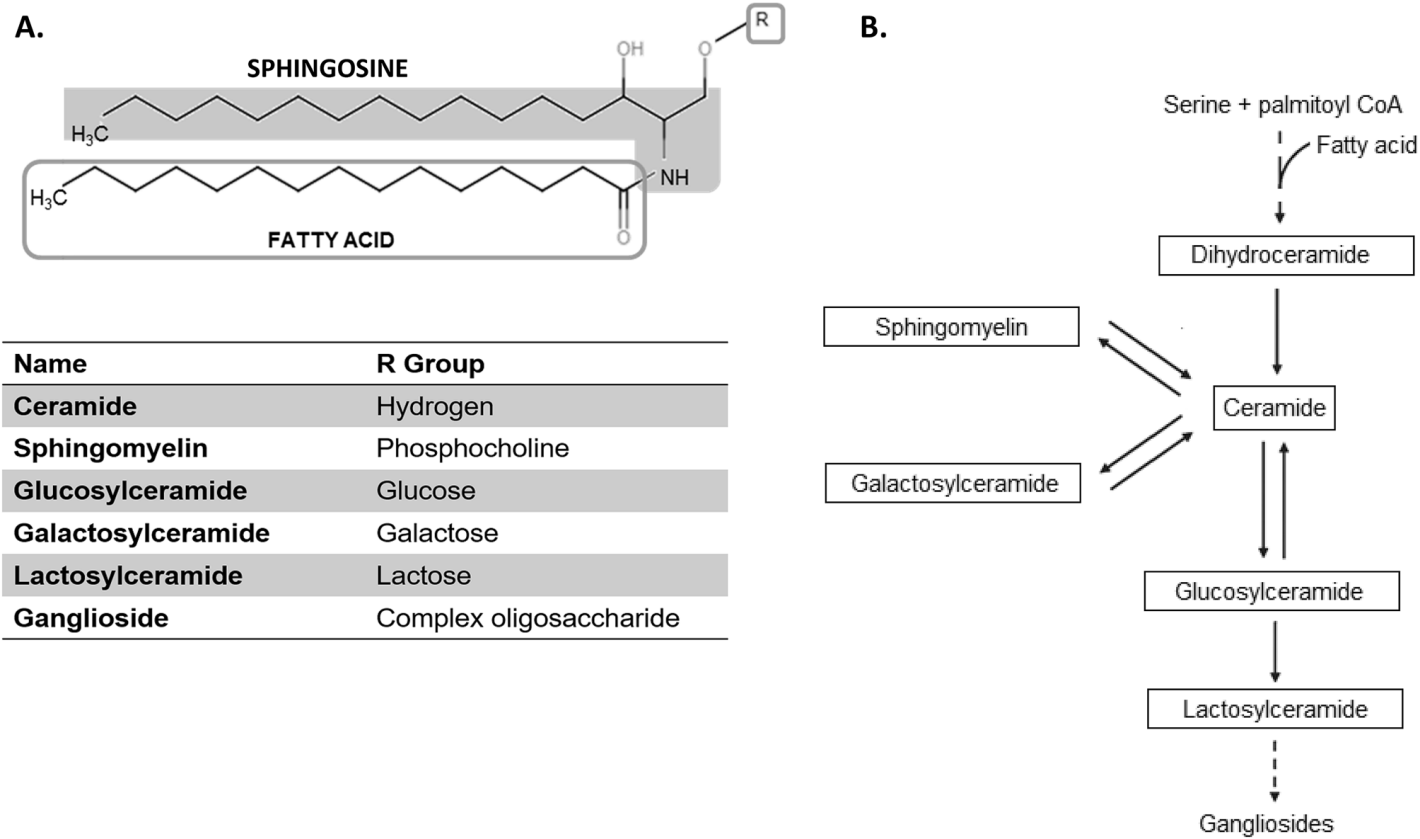
Sphingolipid biochemistry. (**A**) Sphingolipid structure showing the functional group (R) that defines a class and the bound fatty acid that characterizes species based on the number of carbon atoms and double bonds. (**B**) Sphingolipid metabolic pathways. The class referred in the text to as hexosylceramides includes glucosylceramides and galactosylceramides.

Sphingolipids encompass a complex family of circulating and membrane lipids in which a fatty acid is linked to a long sphingosine carbon backbone (Fig. 1). Depending on their head group, they can be divided into different classes (e.g., ceramides, sphingomyelins, hexosylceramides—glucosylceramide or galatosylceramide– and lactosylceramides) (Fig. 1A). Specifically, ceramides lie at the center of the sphingolipid metabolic pathway defined by the oxidation of dihydroceramides or by the interconversion of more complex sphingolipids^14^ (Fig. 1B). Interestingly, sphingolipids are positioned as key signaling molecules regulating a variety of cellular functions and metabolic pathways^15^. Moreover, ceramides have been implicated in several disorders including obesity, diabetes, ASCVD, cancer, and Alzheimer’s disease^16,17^. Further, high levels of ceramides have also been linked to depression^18^, suggesting that abnormal sphingolipid metabolism may also be involved in mental illnesses.

We hypothesized that specific domains of PWB would be associated with a better sphingolipid profile, through restorative health behaviors, formulated as healthy diet or exercise, which lead to improved physiological functioning, reflected by a lower body mass index and better sleep quality. In this study, the relationship between components of eudaimonic well-being and blood sphingolipid levels was examined, with the mentioned health behaviors considered as potential intermediaries of this association.

## Results

### Overall descriptive characteristics of the study sample

The study sample included 967 middle-aged and older adults; over half were women (54.4%) and had some college education (Table 1). More than 50% of the participants were overweight or obese with a median BMI of 28.1 kg/m^2^. With regard to medication use, 34.6% of the participants took anti-hypertensive drugs, 29.6% hypolipidemic agents, 4.2% corticosteroids, and 15.8% were using anti-depressants.

**Table 1:** Descriptive statistics for study variables in participants from MIDUS (n = 967).

Variables	%	Median	Range
Age, years		55	34–84
Sex, % males	45.6		
Education, score points		8	1–12
Anti-hypertensive meds, % yes	34.6		
Cholesterol lowering meds, % yes	29.6		
Corticosteroids, % yes	4.2		
Anti-depressants, % yes	15.8		
BMI, kg/m^2^		28.1	15.0–63.2

**Figure 2:**
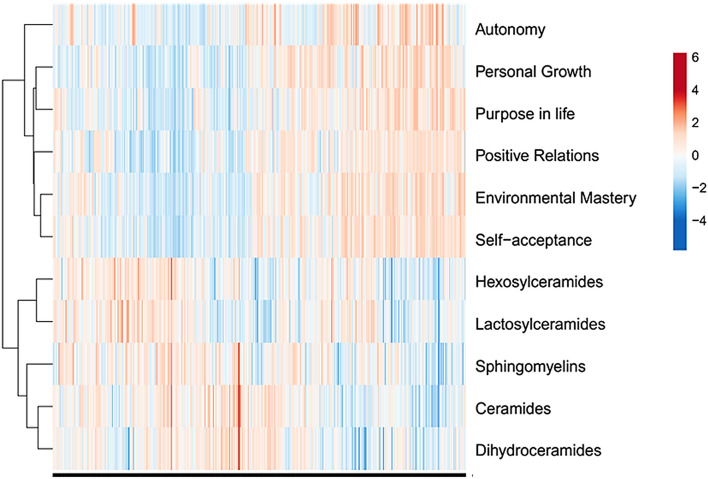
Hierarchical clustering heatmap of subjects from MIDUS, based on PWB dimensions and sphingolipid levels. Columns represent individual participants. Rows represent sphingolipid classes and PWB dimensions. A three-color scale was used with blue indicating low relative values, white indicating average values, and red representing high relative values. This heatmap was performed using the web tool ClustVis 2.0 (https://biit.cs.ut.ee/clustvis).

As an initial approach to visualize the relationship between well-being dimensions and blood sphingolipid classes, a hierarchical log-heatmap clustering analysis was performed (Fig. 2). Overall, the heatmap analysis showed that subjects with higher levels of well-being had lower serum levels of most sphingolipids and vice versa (Fig. 2).

### Inverse associations between eudaimonic well-being dimensions and sphingolipid classes

To further dissect the relationship between serum levels of sphingolipids and eudaimonic well-being dimensions, bivariate analyses were conducted for each of the major variables. As shown in Model 1 (Table 2), higher levels of environmental mastery, purpose in life, and self-acceptance were significantly correlated with lower levels of total ceramides, dihydroceramides, and hexosylceramides. Significant associations were not found for other components of well-being or for other classes of sphingolipids (Table S1).

**Table 2:** Association between blood levels of sphingolipid classes and well-being dimension scores.

	Dihydroceramides		Ceramides		Hexosylceramides	
	Coef	*p*-value	Coef	*p*-value	Coef	*p*-value
**Model 1: Bivariate analysis**						
Environmental mastery	**−** **0.088**	**0.006**	**−** **0.122**	**0.000**	**−** **0.080**	**0.013**
Purpose in life	**−** **0.067**	**0.038**	**−** **0.101**	**0.002**	**−** **0.088**	**0.006**
Self-acceptance	**−** **0.066**	**0.040**	**−** **0.106**	**0.001**	**−** **0.081**	**0.011**
**Model 2: Multivariate analysis adjusting for demographic variables, medication usage and depression**						
Environmental mastery	**−** 0.728	0.059	**−** **0.118**	**0.002**	**−** 0.051	0.152
Purpose in life	**−** 0.023	0.521	**−** 0.531	0.126	**−** 0.059	0.071
Self-acceptance	**−** 0.017	0.657	**−** 0.066	0.079	-0.056	0.109

*p*-values < 0.05 and their standardized regression coefficients are denoted in bold.

The summary statistics for Model 2 (Table 2 and S1) indicate that the significance of some aspects of the associations found between sphingolipids levels and well-being dimensions were modified after adjusting for demographic factors, medication usage, and depression, which should be considered potential covariates. As conveyed by this model, associations between dihydroceramides and hexosylceramides with environmental mastery, purpose in life, and self-acceptance were fully explained by the covariates, particularly by sex and cholesterol medications (Table S2), suggesting that these factors account for the bivariate association (Table 2, Model 2). However, the relationship between ceramides and environmental mastery remained significant even when considering these covariates, including after adjusting for depressive symptoms (assessed by CES-D Scale) (Table 2, Model 2). Therefore, the link between ceramides and environmental mastery did not appear to be dependent on sociodemographic variables, medication use, or statistical biases attributable to negative affect.

### Association between ceramides and environmental mastery: effect of health behaviors

Hierarchical linear regression models were used to further examine the associations between environmental mastery and blood ceramides, and to discern whether health behaviors might be potential mediators of the relationships between PWB and these novel lipids.

Models 3, 4, 5 and 6 examined the role of each health behavior as a possible mediator of the negative association between a sense of more environmental mastery and ceramides (Table 3). Only BMI -as a distal integrative indicator of overall lifestyle- and sleep quality were found to be significantly correlated with ceramide levels. Moreover, the correlation between environmental mastery and ceramide levels remained significant even after adjustments that included the four separate measures of health behavior, suggesting an association that was at least partially independent of these factors (Table 3).

**Table 3:** Multivariate association analysis between environmental mastery and ceramides considering the effect of health behaviors.

	Ceramides vs							
	Coef	*p*-value	Coef	*p*-value	Coef	*p*-value	Coef	*p*-value
Environmental mastery	**−** **0.115**	**0.003**	**−** **0.112**	**0.003**	**−** **0.116**	**0.002**	**−** **0.118**	**0.002**
Diet	**−** 0.041	0.221	**−** 0.035	0.295	**−** 0.030	0.366	**−** 0.032	0.353
Exercise			**−** 0.035	0.285	**−** 0.020	0.549	**−** 0.019	0.573
BMI					**0.113**	**0.001**	**0.116**	**0.001**
Sleep quality							**0.095**	**0.011**

*p*-values < 0.05 and their standardized regression coefficients are denoted in bold.

Models 3, 4, 5 and 6 were also analyzed for other dimensions of well-being and SPL classes. However, no statistically significant associations were found (Table S1). These follow-up analyses also included a seventh model that adjusted for CVD, which could potentially influence the SPL levels. Nevertheless, observed relationships did not lose significance after this additional measure was considered (Table S1).

**Figure 3:**
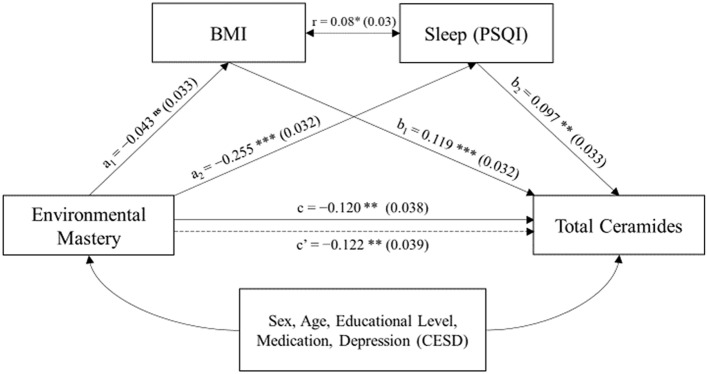
Mediation model of associations between environmental mastery and total ceramides, and possible mediation by sleep quality (PSQI) and BMI. Only participants with data for all variables were included, n = 913. Standardized regression coefficient and standard errors (in parentheses) of the multivariate linear regressions are presented. The paths affecting total ceramides (b and c) controlled for sex, age, educational level, depression (CESD), and medication usage (corticosteroids, cholesterol-lowering, antihypertensive and antidepressants). The direct path coefficient (c’) indicates the effect of environmental mastery on total ceramide levels adjusting for BMI and sleep quality, while the c path coefficient refers to the effect of environmental mastery on total ceramides without adjustments for mediators. The r value indicates Pearson correlation coefficient between BMI and sleep (PSQI). *p < 0.05, **p < 0.01, ***p < 0.001. ^ns^, not significant.

Subsequently, mediation bootstrapping analyses were conducted to statistically confirm the mediating effect of BMI and sleep quality on the relationship between environmental mastery and total ceramides. As observed in the mediation model (Fig. 3**)** and concurring with the hierarchical analysis (Table 3), both BMI and quality of sleep significantly affected blood ceramide levels. However, based on the bootstrapping method, only sleep quality was a statistically significant mediator of the relationship between environmental mastery and total ceramides, when considering both parameters as independent mediators (Table 4). Nevertheless, when the joint effect of both mediators was considered as representative overall health behaviors, the mediation was statistically significant (Table 4).

**Table 4:** Bootstrapping to test indirect and total effects of environmental mastery on blood ceramide levels that were mediated by sleep quality (PSQI) and BMI, separately or combined.

Effects	Coef, ± SE	95% CI	p-value
**Indirect effect**			
Sleep (PSQI)	**−** 0.024 ± 0.011	**−** 0.046, **−** 0.004	0.022
BMI	**−** 0.005 ± 0.005	**−** 0.016, 0.003	ns
Both	**−** 0.030 ± 0.011	**−** 0.053, **−** 0.008	0.008
**Total effect**			
Total	**−** 0.152 ± 0.038	**−** 0.229, **−** 0.078	< 0.001

### Association between environmental mastery and sphingolipid species

Within each sphingolipid class, there are many component species defined by structural and chemical features (i.e., carbon chain length, double bonds) of the attached fatty acids (Fig. 1A) that correlate with different functions and targets^17^. Because the levels of each species can also differ^17^, the same hierarchical models were applied to more specifically delineate the relationship between each molecular species of sphingolipids and the different dimensions of eudaimonic well-being (Table 5).

**Table 5:**
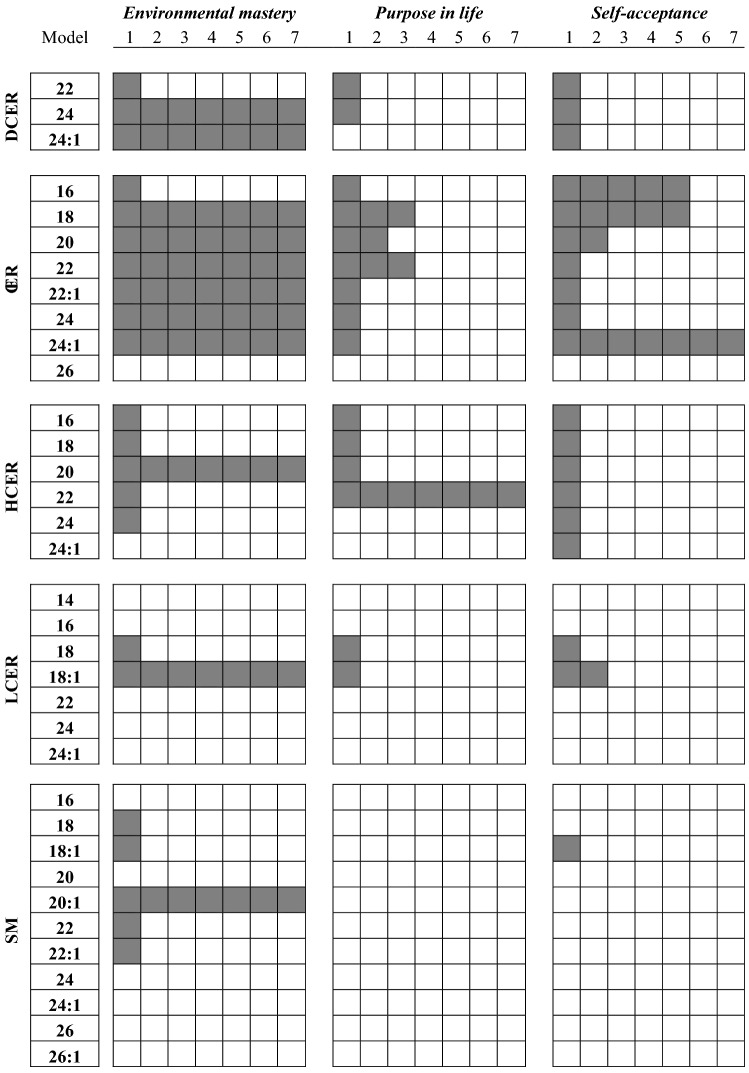
Multivariate association analysis of the relationship between psychological well-being domains and individual species of sphingolipids.

Model 1: bivariate analysis. Model 2: adjusted for demographic factors, medication usage, and depression. Models 3, 4, 5 and 6 determined the influence of each health behavior as a possible mediators, adding them sequentially: food intake quality (MIDUS-HEI) in Model 3, exercise (METs) in Model 4, sleep quality (PSQI) in Model 5, and Body Mass Index (BMI, kg/m^2^) in Model 6. Model 7 includes adjustments for CVD and diabetes.

Gray boxes represent statistically significant associations (all with *p* < 0.05 and regression coefficients < 0).

Based on the hierarchical analysis shown for Model 1, all three dihydroceramides included in this analysis were negatively correlated with environmental mastery. DCER24 and DCER24:1 remained significantly associated with environmental mastery after adjustments by the covariates in Model 2. Models 3, 4, 5, and 6 indicated these relationships were partially attenuated by BMI (based on β reduction and *p* < 0.05 for BMI), but remained significant after full adjustments (Table 5).

Similarly, six out of seven species of ceramides exhibited negative and statistically significant correlations with environmental mastery (Table 5). Considering these six ceramide species, only the correlation observed with CER16 lost significance after being adjusted for the covariates in Model 2. Interestingly, Models 3, 4, 5, and 6 indicated that all health behaviors were significantly associated with ceramides at the species level of analysis (*p* < 0.05 for each health behavior). Nonetheless, the fully adjusted model demonstrated that the association between these ceramide species and environmental mastery remained significant (Table 5).

Some hexosylceramide, lactosylceramide and sphingomyelin species showed significant inverse correlations with environmental mastery (Table 5). However, only the associations between HCER20, LCER18:1 and SM20:1 with environmental mastery remained significant after adjustments for covariates (Table 5). The health behaviors included in Models 3, 4, 5, and 6 did not appear to exert any significant influence on these latter associations (Table 5).

### Association between purpose in life and self-acceptance with sphingolipid species

Six species of ceramides (CER16, 18, 20, 22, 24, and 24:1) were negatively correlated with purpose in life and self-acceptance (Table 5). Only three (CER18, CER20, and CER22) were significantly associated with purpose in life after the covariate adjustments. In contrast, 4 ceramides (CER16, 18, 20, and 24:1) evidenced a significant relationship with self-acceptance after adjustments (Model 2). In all cases, the associations between these two PWB domains were attenuated to non-significant levels when looking at the species level of ceramides after adjusting for health behaviors (Models 3, 4, 5, and 6). This outcome suggested that the observed correlations were more appropriately explained by health behaviors. Similarly, most of the dihydroceramide and hexosylceramide species appeared initially to have a negative relationship with purpose in life and self-acceptance. However, these correlations no longer attained statistical significance after the covariate adjustments (Table 5). The only exception was HCER22, which remained significantly associated with purpose in life in the hierarchical models after inclusion of health behaviors. Only a few individual species of the sphingomyelins and lactosylceramides had significant associations with these two PWB domains, and all became nonsignificant after adjustments (Table 5). The same models were performed for other PWB domains (autonomy, personal growth and positive relations). However, no species showed significant associations after adjusting for control variables.

## Discussion

PWB has been linked with a range of health outcomes^2,6^, but the evidence connecting PWB to primary underlying biological processes has been limited. The current analyses demonstrated significant associations between eudaimonic well-being and serum sphingolipid profile in middle-aged and older white adults in a national US sample. In particular, environmental mastery was correlated with lower levels of total ceramides and most of the assessed ceramide species in cross-sectional models, after controlling for relevant demographic factor, medication usage, and depression. In addition, purpose in life and self-acceptance were correlated with lower levels of specific ceramide species. In recent papers, high ceramide levels have been reported in pathological conditions including diabetes, coronary artery disease, as well as depression^17^. Ceramides may thus constitute a biological intermediary between PWB and a number of health outcomes in adults. Of particular relevance, our analyses considered daily health practices as potential pathways through which well-being might influence a person’s sphingolipid profile. We found that nutritional status and sleep quality can modulate and contribute to the observed associations.

Previous studies have connected positive psychosocial factors to healthy routine lipid profiles. Hedonic well-being and optimism were correlated with higher HDL-cholesterol levels and lower triglycerides levels^19,20^. However, studies linking eudaimonic well-being and other lipids are limited. It is accepted that life engagement requires a person to be active and striving^21^. As such and distinct from other formulations of wellbeing, eudaimonic well-being may directly stimulate protective and restorative processes directly or indirectly through promoting healthy behaviors. Indeed, we have shown that persistently high levels of eudaimonic well-being were predictive of a better blood lipid profile. Specifically, persistently high levels of environmental mastery and self-acceptance predicted high HDL cholesterol and low triglycerides^13^. Considering the associations shown in the present study, these two eudaimonic features seem to have a stronger impact on lipid metabolism than other dimensions.

Previously hypothesized mediators between well-being and reduced CVD include better autonomic and neuroendocrine regulation, reduction of inflammatory mediators, and improved metabolic functioning^2^. The sphingolipids appear to be another key link between well-being and health. Emerging lipidomic approaches have generated a large body of evidence demonstrating that sphingolipids are signaling molecules that regulate a diverse range of cellular processes in several pathophysiological conditions, such as inflammation and apoptosis^17^. In fact, ceramide modulation may underlie the pathogenesis of a wide variety of diseases, including insulin resistance, diabetes, atherosclerosis, neurodegenerative disorders, and cancer^17^. Therefore, the strong negative association between eudaimonic well-being indicators and ceramide levels observed in this study has significant implications for disease risk and pathogenesis.

We also found a negative association between environmental mastery and some dihydroceramide species. Dihydroceramides are the precursors of de novo synthesis of ceramides. While the pathophysiological effects of dihydroceramides are still controversial, they have recently been considered to be indicators of metabolic dysfunction^22^. In addition, hexosylceramides were negatively correlated with some dimensions of well-being, after adjusting for control variables. Hexosylceramides (i.e., glucosylceramides and galactosylceramides) have been associated with some protective effects^23^, although also with cancer and inflammatory disorders^24,25^.

Interestingly, sphingolipids have been also involved in the regulation of aging and senescence in multiple cell types and experimental organisms^26^. Although further mechanistic research on bioactive sphingolipids and aging is needed, ceramides are known to be involved in cellular senescence and death as well as age-related diseases. Thus, the inverse link between eudaimonic measures and ceramide levels may help explain the positive associations between well-being and healthy aging. The importance of eudaimonic wellbeing has become more prominent beyond specific diseases extending to quality of life, a sense of vitality and longevity^27^.

The strong negative association between ceramides and environmental mastery was partly mediated by health behaviors, specifically by BMI and sleep quality. In prior work, PWB has been associated with restorative health behaviors^7^, such as physical activity^9^, healthy eating^11^, and good sleep^10^. Both physical activity and a healthy diet pattern may affect ceramide levels. Western diet consumption and obesity are known to increase circulating ceramide levels^28–30^. In addition, ceramide levels in muscle are reduced following exercise training^31^. Even though the contribution of diet and exercise was modest in our study, it could be explained by high collinearity of both variables with nutritional status. Thus, only BMI was added in the mediation analysis between environmental mastery and ceramide levels. According to bootstrapping, sleep quality emerged as a statistically significant mediator, but not BMI. However, the effect of environmental mastery on blood ceramide levels through BMI and sleep quality together was statistically significant and higher than the independent indirect effects. Considering the low collinearity of both parameters (r < 0.1), this result suggests that these indicators participate jointly in the mediation of environmental mastery and ceramide levels. If so, improving sleep quality and BMI may reduce serum ceramide levels and enhance the effect of environmental mastery. Interestingly, sleep quality was the most relevant intermediary among various behaviors analyzed here, although little is known about the relationship between sleep and ceramides. However, poor sleep quality disturbs the circadian rhythm, altering metabolic, inflammatory and neuroendocrine biomarkers^32^, which may eventually play a role in increasing ceramide levels.

The significant negative correlation between ceramides and environmental mastery in the fully adjusted model suggests that the relationship partially depends on the studied pathways. The reverse may also be true: high levels of ceramides may negatively affect brain processes, leading to worsened PWB measures. If so, inducible dysfunction of the ceramide pathway may account for mood disorders and behavioral abnormalities^33^. Indeed, increased plasma ceramide levels have been associated with depressive symptoms in humans and animal models^33,34^. The cross-sectional design of this study prohibits inferring on the causal direction of the association between eudaimonic well-being and ceramides, which need to be studied longitudinally. Interestingly, a recent study found changes in several phospholipids species after a lifestyle intervention aimed to improve well-being^35^. The associations suggested the involvement of less well-studied lipids in important pathways underlying psychological and metabolic health^35^.

The present study has several strengths, including sampling a relatively large number of American adults from the 48 continental states. The availability of lipidomic data and information on many contributing factors and potentially confounding variables were also unique On the other hand, the analyses were limited to middle-aged and older adults of one race limiting the generalizability of our findings. Additional analyses are currently being conducted in the African American participants from MIDUS, given the known differences in lipid metabolism and health^36^. In addition, single time point for assessing lipids precluded conclusions about the directionality of the effects. It should also be acknowledged that the lipid data were generated with an untargeted approach, designed for the discovery of relationship. Further analysis will be required to confirm the quantification of the lipid species found to be a low concentration. However, the main conclusions of this study were generated from ceramides, a well-represented class.

In conclusion, we identified a significant association of eudaimonic well-being on blood sphingolipid profiles in caucasian American adults, partially mediated by their health behaviors. This association affirms the promise for lifestyle and behavioral interventions that induce favorable sphingolipid changes via an enhancement of eudaemonic well-being, or conversely, that facilitate health-promoting behaviors and dietary changes that modify sphingolipids in ways that result in healthier aging.

## Methods

### Sample

The MIDUS (Midlife in the US) study was initiated in 1995/96 to investigate the role of behavioral, psychological, and social factors on physical and mental health across adult life. All eligible participants were non-institutionalized, English-speaking, 25–74 years-old adults living in the United States. A follow-up (MIDUS 2) was conducted approximately 9–10 years later^37^. In addition, comprehensive biomarker evaluations were obtained on a subsample of participants (n = 1255). Of those individuals who participated in biomarker assessments, we excluded 35 who did not have lipidomic data as well as the small subsample of African Americans, given that plasma sphingolipid levels and their pattern in health disorders are known to be distinct between blacks and whites^38,39^. Thus, current analyses were based on the white subpopulation of MIDUS with a final subsample of 967 participants.

### Ethics

Specimen collection and testing were approved by the Health Sciences Institutional Review Board at the University of Wisconsin-Madison, as well as by the Institutional Review Boards at the University of California-Los Angeles and Georgetown University. The study was conducted in accordance with the Declaration of Helsinki and all participants gave written informed consent. All data were handled anonymously.

### Measures

#### Eudaimonic well-being

Eudaimonic well-being measurements were based on Ryff’s scales to evaluate six components: autonomy, environmental mastery, personal growth, positive relationships, purpose in life, and self-acceptance^4,39^. The six psychological well-being subscales each represented the respective 7-item mean of responses to a 7-point Likert-type scale^37^. Briefly: *Autonomy* emphasizes that one is self-determining and independent as well as able to evaluate oneself by personal standards. *Environmental mastery* emphasizes the sense that one can manage the surrounding environment, including making effective use of available opportunities. *Personal growth* is concerned with self-realization and achievement of personal potential. *Positive relations with others* encompass having warm, trusting ties to others, being concerned about the welfare of others and understanding the give and take of social relationships. *Purpose in life* emphasizes on viewing one’s life has having meaning, direction, and goals. Finally, *self-acceptance* encompasses having positive attitudes toward oneself, but also includes the capacity to see one’s bad qualities^4,39^.

#### Sphingolipid profile

Serum sphingolipid profiling was performed as part of an untargeted lipidomic approach by Metabolon, Inc. (Durham, NC), following the protocol described in Supplementary materials. For our study, only sphingolipid values, i.e., dihydroceramides, ceramides, sphingomyelins, hexosylceramides -encompassing glucosylceramides and galactosylceramides, and lactosylceramides were used. Before statistical analyses, sphingolipid levels were log_e_-transformed to achieve normal distributions and normalized using z-score. Only those species with < 20% of missing values (due to levels below the lowest limit of detection) were included in the statistical modeling.

#### Control variables

Sociodemographic variables and information on medication use was obtained during the survey and at the time of blood collection. Demographic factors included age (continuous), sex (0 = male, 1 = female), and level of educational attainment (from 1 = no school to 12 = PhD, MD, or other professional degree). Medication use included drugs for hypertension, dyslipidemia, steroids or anti-depressants being taken at the time of blood collection. In addition, the potential influence of depression was considered using log-transformed values of the Center for Epidemiological Studies Depression (CES-D) scale^40^. Finally, self-report of previous medical diagnosis of heart disease, stroke, or transient ischemic attack was used as indicator of CVD.

#### Health behaviors

Health behaviors included food intake quality, exercise, and sleep quality. To assess quality of food consumption, a healthy diet index was used, which categorized respondents from 0 (unhealthy diet) to 11 (healthy diet) points by evaluating intake of sugary beverages, vegetables and fruits, whole grains, fish, fat meat, lean meat, non-meat protein, fast food, alcohol, and fermented dairy products (Echeverría et al., in preparation). To estimate physical activity, the amount of energy spent doing exercise was calculated as metabolic equivalent for task (MET)^41^. Sleep quality was assessed using the Pittsburgh Sleep Quality Index (PSQI) by which lower scores denote healthier sleep^42^ after integrating subjective sleep quality, sleep latency and duration, sleep efficiency, sleep disturbances, use of sleeping medication, and daytime dysfunction. Body mass index (BMI, kg/m^2^) was also used as a surrogate distal indicator of overall lifestyle behaviors.

### Data analysis

#### Heatmap

As an initial exploratory analysis, a heatmap of eudaimonic well-being dimensions and sphingolipid classes was performed using the web tool ClustVis 2.0^43^ (https://biit.cs.ut.ee/clustvis). Each element of the matrix represents the fold-change obtained by a subject (column) for a specific variable (row), in relation to total sample average. Fold-changes were log2-transformed, thus positive values (red) show levels above average, while negative values (blue) indicate levels below average. Both rows and columns were clustered using correlation distance and average linkage.

#### Statistical analyses

To evaluate an association between well-being domains and sphingolipid levels, we conducted a series of hierarchical multiple regression analyses for each well-being dimension and sphingolipid class (or species). Prior steps of data transformation were required: eudaimonic dimension scales were cubed to symmetrize the distribution of scores whereas depression (CES-D) scale, metabolic equivalent for task (MET), and serum sphingolipid levels were log_e_-transformed to achieve normal distributions. All other variables were mean-centered and did not require transformation. Finally, all data were normalized by z-scores before the statistical analysis.

Hierarchical multivariate analyses were conducted sequentially as follows. First, in Model 1, bivariate associations between a well-being dimension and a sphingolipid class were explored. The second model (Model 2) added adjustments for demographic factors (i.e., age, sex, education), medication use, and depression. Models 3, 4, 5, and 6 were conducted to evaluate the role of each health behaviors (food quality, exercise, sleep quality, and BMI) as potential mediators for the associations between well-being and sphingolipid levels: Model 3 included food quality intake (healthy diet index), Model 4 added exercise (energy expenditure), Model 5 considered BMI and, finally, Model 6 took into account sleep quality. Finally, to rule out that the relationship between well-being and serum SPL levels was explained by previous cardiovascular conditions, model 7 added adjustments for CVD and diabetes. The hierarchical order was defined by the physiological relationship between adjustment variables.

To investigate health behaviors as potential mediators of the relationship between PWB and blood sphingolipid levels, percentile bootstrapping-based mediation analysis was finally performed^44–46^ using those variables that resulted statistically significant in hierarchical regression analysis. If 0 landed outside of the confidence interval (CI), mediation was likely, whereas if 0 was within the CI, there was no statistical evidence for mediation. Mean indirect and total effects were computed across 5000 bootstrap samples to represent the final indirect effect estimate.

Statistical analyses were conducted using R-studios Desktop, version 1.2.5001. All *p* values were two-tailed and considered statistically significant when *p* ≤ 0.05.

## Supplementary Information

Supplementary Information.

## Data Availability

Most of the data used in this research is available through the study-wide public portal located at https://midus.colectica.org/. Lipidomic data was deposited to OSF database under Accession Name LipPWB3210391 (https://osf.io/vfr7b/?view_only=a784e7d6427a4583a90c21d94a73fe74).
