# Supplementary material for: Association between serum sphingolipids and eudaimonic well-being in white U.S. adults
Source: Sci Rep. 2021 Jun 23;11:13139. doi: 10.1038/s41598-021-92576-3 (PMC8222370; doi:10.1038/s41598-021-92576-3)
Supplement: Supplementary file 1 — Supplementary Information. [file 41598_2021_92576_MOESM1_ESM.docx]

**Association Between Serum Sphingolipids and Eudaimonic Well-Being in White U.S. Adults**

Loni Berkowitz^1*^, Marcela P Henríquez^2^, Cristian Salazar^1^, Eric Rojas^3^, Guadalupe Echeverría^1^,

Gayle D Love^4^, Attilio Rigotti^1^, Christopher L Coe^4^, and Carol D Ryff^4^

^1^Department of Nutrition, Diabetes and Metabolism, Center of Molecular Nutrition and Chronic Diseases, School of Medicine, Pontificia Universidad Católica de Chile, Santiago, Chile.

^2^ELSA Clinical Laboratory, IntegraMedica, Bupa. Santiago, Chile.

^3^Department of Clinical Laboratories, School of Medicine, Pontificia Universidad Católica de Chile, Santiago, Chile.

^4^Institute on Aging, University of Wisconsin-Madison, Madison, Wisconsin, USA.

**Keywords:** eudaemonic well-being, sphingolipids, ceramides, health behaviors.

***Corresponding author**: Loni Berkowitz, Center of Molecular Nutrition and Chronic Diseases,

Department of Nutrition, Diabetes and Metabolism, School of Medicine, Pontificia Universidad Católica de Chile, Marcoleta 328, Santiago, Chile. E-mail: loniberko@gmail.com

**Supplementary Information**

**Lipid extraction and quantification**

Lipid profiling was performed as part of an untargeted-lipidomic approach by Metabolon, Inc. (Durham, NC), following the protocol described below.

- Extraction: Lipids were extracted from the bio-fluid in the presence of deuterated internal standards using an automated BUME extraction according to the method of Lofgren et al^40^.
- Data Acquisition: The extracts were dried under nitrogen and reconstituted in ammonium acetate dichloromethane:methanol. The extracts were transferred to vials for infusion-MS analysis, performed on a Shimadzu LC with nano PEEK tubing and the Sciex SelexIon-5500 QTRAP. The samples were analyzed via both positive and negative mode electrospray. The 5500 QTRAP was operated in multiple reaction monitoring (MRM) mode with a total of more than 1,100 MRMs.
- Quantification: Quantitation was built using class-specific internal standards with each class containing at least one, with most containing multiple labeled internal standards. Individual lipid species were quantified by taking the ratio of the signal intensity of each target compound to that of its assigned internal standard, then multiplying by the concentration of internal standard added to the sample (e.g. dCER (d16:0) and dDCER(16:0) for ceramides and dihydroceramides, respectively). Lipid class concentrations were calculated from the sum of all molecular species within a class, and fatty acid compositions were determined by calculating the proportion of each class comprised by individual fatty acids. The quantification of the different species and classes of lipids is reported as micromolar concentration. For our study, we only used sphingolipid values (dihydroceramides, ceramides, sphingomyelins, hexosylceramides -encompassing glucosylceramides and galactosylceramides, and lactosylceramides).
- Data processing: Before statistical analyses, sphingolipid levels were log_e_-transformed to achieve normal distributions and normalized using z-score. Only those species with <20% of missing values (due to levels below the lower limit of detection) were included in the statistical modeling.

**Table S1. Multivariate association analysis of the relationship between psychological well-being domains and sphingolipid classes.**

|  | **DCER** | **CER** | **HCER** | **LCER** | **SM** |
| --- | --- | --- | --- | --- | --- |
|  | *Coef.* | *Coef.* | *Coef.* | *Coef.* | *Coef.* |
| ***Autonomy*** |  |  |  |  |  |
| Model 1 | 0.032 | -0.014 | -0.030 | -0.012 | -0.030 |
| Model 2 | 0.045 | -0.007 | 0.001 | 0.015 | 0.019 |
| Model 3 | 0.048 | -0.003 | 0.002 | 0.015 | 0.016 |
| Model 4 | 0.051 | -0.001 | 0.002 | 0.012 | 0.012 |
| Model 5 | 0.037 | -0.008 | 0.009 | 0.020 | 0.008 |
| Model 6 | 0.046 | -0.003 | 0.013 | 0.016 | 0.006 |
| Model 7 | 0.049 | 0.002 | 0.017 | 0.017 | 0.011 |
| ***Environmental mastery*** |  |  |  |  |  |
| Model 1 | **-0.088** | **-0.122** | **-0.080** | -0.005 | -0.060 |
| Model 2 | -0.073 | **-0.118** | -0.051 | 0.017 | -0.018 |
| Model 3 | -0.070 | **-0.115** | -0.052 | 0.018 | -0.019 |
| Model 4 | -0.067 | **-0.112** | -0.052 | 0.013 | -0.024 |
| Model 5 | -0.075 | **-0.116** | -0.048 | 0.018 | -0.027 |
| Model 6 | -0.076 | **-0.118** | -0.054 | 0.009 | -0.028 |
| Model 7 | -0.075 | **-0.115** | -0.049 | 0.011 | -0.025 |
| ***Personal growth*** |  |  |  |  |  |
| Model 1 | -0.039 | -0.052 | -0.016 | 0.035 | 0.039 |
| Model 2 | 0.015 | 0.001 | 0.007 | 0.032 | 0.036 |
| Model 3 | 0.018 | 0.008 | 0.010 | 0.036 | 0.034 |
| Model 4 | 0.021 | 0.012 | 0.011 | 0.032 | 0.029 |
| Model 5 | 0.024 | 0.013 | 0.010 | 0.031 | 0.029 |
| Model 6 | 0.024 | 0.015 | 0.008 | 0.022 | 0.029 |
| Model 7 | 0.025 | 0.018 | 0.010 | 0.023 | 0.032 |
| ***Positive relations*** |  |  |  |  |  |
| Model 1 | -0.037 | -0.030 | -0.058 | -0.018 | 0.045 |
| Model 2 | 0.012 | 0.026 | -0.017 | -0.018 | 0.045 |
| Model 3 | 0.017 | 0.032 | -0.015 | -0.017 | 0.043 |
| Model 4 | 0.018 | 0.034 | -0.015 | -0.019 | 0.041 |
| Model 5 | 0.010 | 0.029 | -0.010 | -0.014 | 0.038 |
| Model 6 | 0.011 | 0.031 | -0.013 | -0.023 | 0.034 |
| Model 7 | 0.011 | 0.032 | -0.014 | -0.023 | 0.034 |
| ***Purpose in life*** |  |  |  |  |  |
| Model 1 | **-0.067** | **-0.100** | **-0.089** | -0.010 | -0.021 |
| Model 2 | -0.023 | -0.053 | -0.059 | 0.003 | 0.006 |
| Model 3 | -0.019 | -0.046 | -0.057 | 0.005 | 0.004 |
| Model 4 | -0.016 | -0.043 | -0.057 | 0.002 | 0.000 |
| Model 5 | -0.015 | -0.043 | -0.057 | 0.001 | 0.000 |
| Model 6 | -0.017 | -0.038 | -0.057 | -0.005 | -0.002 |
| Model 7 | -0.017 | -0.037 | -0.057 | -0.005 | -0.002 |
| ***Self*-*acceptance*** |  |  |  |  |  |
| Model 1 | **-0.066** | **-0.106** | **-0.081** | -0.015 | -0.051 |
| Model 2 | -0.017 | -0.066 | -0.056 | -0.003 | -0.008 |
| Model 3 | -0.013 | -0.061 | -0.054 | -0.002 | -0.010 |
| Model 4 | -0.011 | -0.059 | -0.054 | -0.004 | -0.012 |
| Model 5 | -0.017 | -0.062 | -0.051 | -0.001 | -0.014 |
| Model 6 | -0.015 | -0.057 | -0.052 | -0.009 | -0.015 |
| Model 7 | -0.013 | -0.053 | -0.047 | -0.007 | -0.011 |

*Model 1: bivariate analysis. Model 2: adjusted for demographic factors, medication usage, and depression. Models 3, 4, 5 and 6 determined the influence of each health behavior as a possible mediators, adding them sequentially: food intake quality (MIDUS-HEI) in Model 3, exercise (METs) in Model 4, sleep quality (PSQI) in Model 5, and Body Mass Index (BMI, kg/m^2^) in Model 6. Model 7 includes adjustments for CVD.

*Gray boxes represent statistically significant associations (all with p<0.05 and correlation coef<0).

**Table S2**. **Multivariate analysis of significant associations between sphingolipid classes and well-being dimensions, adding demographic variables, medication usage, and depression.**

|  | **DCER** | |  | **CER** | | | **HCER** | |
| --- | --- | --- | --- | --- | --- | --- | --- | --- |
|  | ***Coef.*** | ***p-value*** |  | ***Coef.*** | ***p-value*** |  | ***Coef.*** | ***p-value*** |
|  |  |  |  |  |  |  |  |  |
| **Environmental mastery** | -0.073 | 0.059 |  | **-0.118** | **0.002** |  | -0.051 | 0.152 |
| Age | 0.038 | 0.294 |  | **0.124** | **0.001** |  | 0.030 | 0.367 |
| Sex | **-0.253** | **<0.001** |  | **-0.270** | **<0.001** |  | -0.105 | 0.085 |
| Education | -0.054 | 0.097 |  | -0.045 | 0.164 |  | -0.035 | 0.248 |
| Blood pressure med. | **-0.242** | **0.001** |  | -0.079 | 0.284 |  | **0.256** | **0.0002** |
| Cholesterol med. | **0.231** | **0.003** |  | **0.397** | **<0.001** |  | **0.791** | **<0.001** |
| Corticosteroids | 0.136 | 0.388 |  | 0.076 | 0.626 |  | 0.096 | 0.516 |
| Antidepressants | -0.095 | 0.289 |  | -0.131 | 0.140 |  | -0.003 | 0.971 |
| Depression | 0.040 | 0.295 |  | 0.059 | 0.115 |  | 0.057 | 0.104 |
|  |  |  |  |  |  |  |  |  |
| **Purpose in life** | -0.023 | 0.521 |  | -0.053 | 0.1263 |  | -0.059 | 0.071 |
| Age | 0.0222 | 0.527 |  | **0.099** | **0.004** |  | 0.019 | 0.553 |
| Sex | **-0.250** | **<0.001** |  | **-0.262** | **<0.001** |  | -0.097 | 0.111 |
| Education | -0.057 | 0.084 |  | -0.047 | 0.143 |  | -0.034 | 0.264 |
| Blood pressure med. | **-0.248** | **<0.001** |  | -0.088 | 0.234 |  | **0.252** | **<0.001** |
| Cholesterol med. | **0.225** | **0.004** |  | **0.388** | **<0.001** |  | **0.785** | **<0.001** |
| Corticosteroids | 0.140 | 0.376 |  | 0.079 | 0.612 |  | 0.092 | 0.533 |
| Antidepressant | -0.100 | 0.266 |  | -0.137 | 0.124 |  | -0.004 | 0.957 |
| Depression | 0.066 | 0.065 |  | **0.094** | **0.008** |  | 0.058 | 0.081 |
|  |  |  |  |  |  |  |  |  |
| **Self-acceptance** | -0.017 | 0.657 |  | -0.066 | 0.079 |  | -0.056 | 0.109 |
| Age | 0.024 | 0.491 |  | **0.108** | **0.002** |  | 0.027 | 0.414 |
| Sex | **-0.252** | **<0.001** |  | **-0.264** | **<0.001** |  | -0.100 | 0.101 |
| Education | -0.056 | 0.089 |  | -0.043 | 0.185 |  | -0.031 | 0.309 |
| Blood pressure med. | **-0.245** | **0.001** |  | -0.078 | 0.295 |  | 0.261 | **<0.001** |
| Cholesterol med. | **0.226** | **0.004** |  | **0.388** | **<0.001** |  | **0.786** | **<0.001** |
| Corticosteroids | 0.140 | 0.374 |  | 0.076 | 0.626 |  | 0.091 | 0.537 |
| Antidepressants | -0.099 | 0.275 |  | -0.129 | 0.147 |  | 0.002 | 0.984 |
| Depression | 0.067 | 0.072 |  | **0.085** | **0.021** |  | 0.056 | 0.110 |

* p-values <0.05 and their standardized regression coefficients are denoted in bold.
